# Supplementary material for: Multiplexed 3D super-resolution imaging of whole cells using spinning disk confocal microscopy and DNA-PAINT
Source: Nat Commun. 2017 Dec 12;8:2090. doi: 10.1038/s41467-017-02028-8 (PMC5727263; doi:10.1038/s41467-017-02028-8)
Supplement: Supplementary file 1 — Supplementary Information [file 41467_2017_2028_MOESM1_ESM.pdf]

# Multiplexed 3D super-resolution imaging of whole cells using Spinning Disk Confocal Microscopy and DNA-PAINT

Florian Schueder, Juanita Lara-Gutiérrez, Brian J Beliveau, Sinem K Saka, Hiroshi M Sasaki, Johannes B Woehrstein, Maximilian T Strauss, Heinrich Grabmayr, Peng Yin and Ralf Jungmann

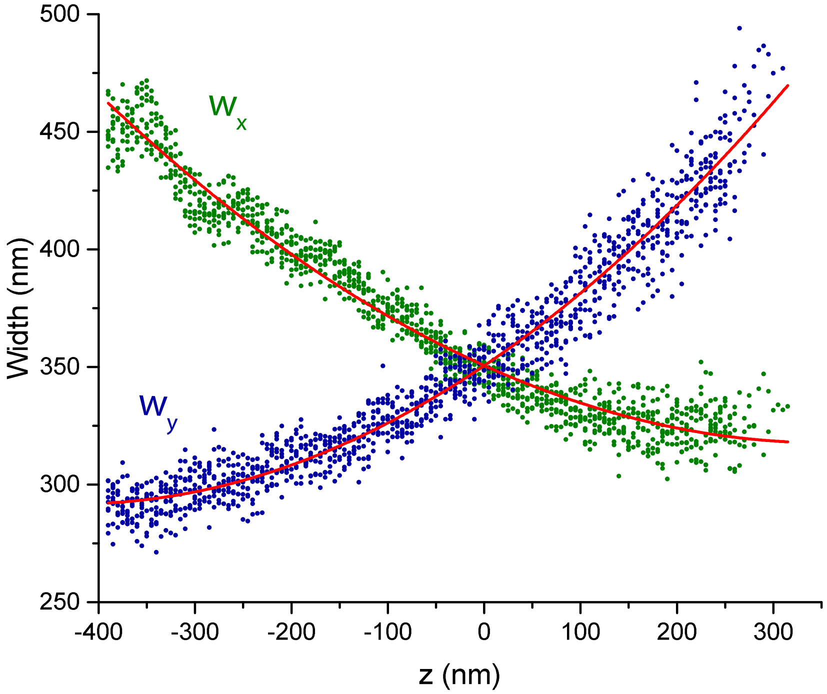


**Supplementary Figure 1 | Calibration data with second degree polynomial fit for 3D super-resolution experiments.** Width of x position (green) and width of y (blue) position data of a fluorescent bead sample plotted with respect to the z position of the beads. In red: second degree polynomial fit of the data which was used to calibrate the absolute z position of the 3D super-resolution experiments.

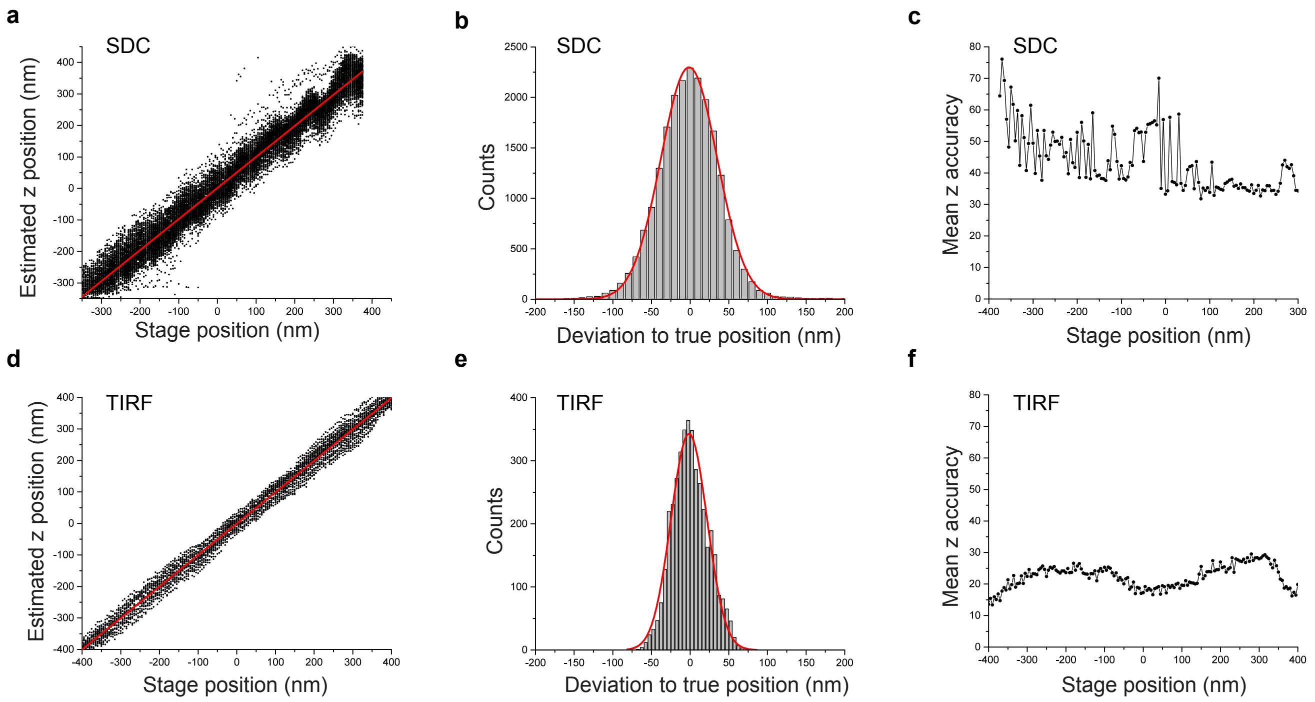


**Supplementary Figure 2 | Accuracy of z-position measurement for SDC and TIRF obtained from a bead sample.** (**a**) Estimated z position of fluorescent beads with respect to stage position. The red line represents a linear fit of the data (black). The slope of the linear fit was determined to be 0.986. (**b**) Histogram representation of the distance of the estimated z position for each bead from the true position (stage position). Histogram fit (red) with a Gaussian function ($\sigma$ = 37 nm). (**c**) Mean z-position accuracy vs. stage position. (**d–f**) Corresponding plots for TIRF. (**d**) The slope of the linear fit was determined to be 0.998. (**e**) Histogram fit (red) with a Gaussian function ($\sigma$ = 23 nm). (**f**) Mean z-position accuracy vs. stage position.

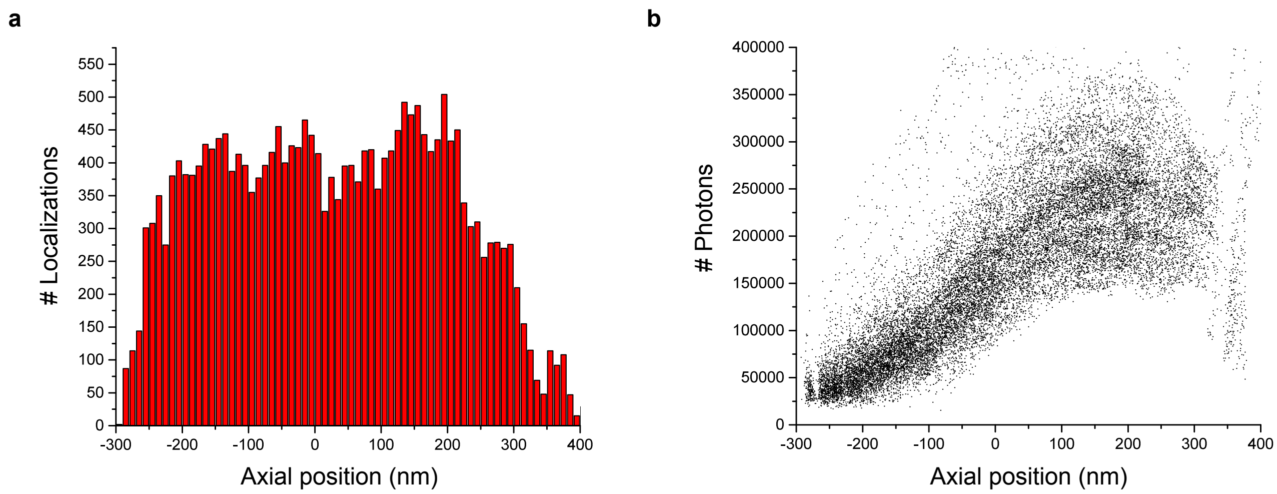


**Supplementary Figure 3 | Detected number of localizations and photons of a bead sample through the field of depth.** (**a**) Number of detected localizations with respect to the axial position (z position) throughout a confocal volume. (**b**) Number of photons per localization vs axial position.

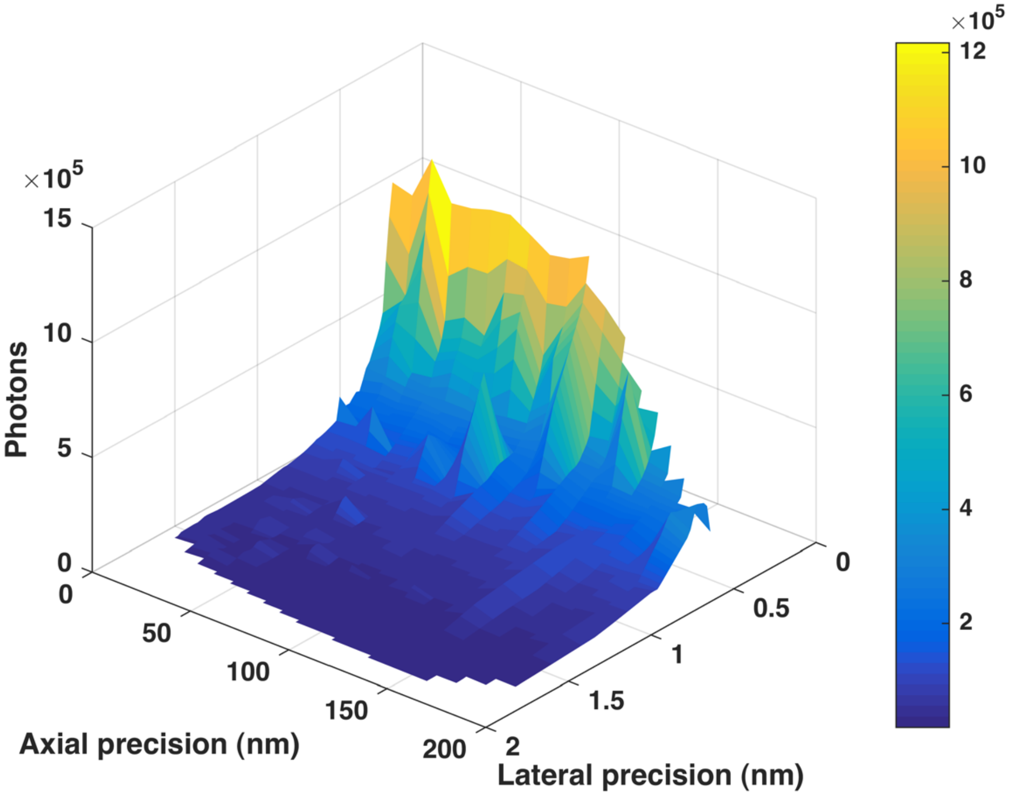


**Supplementary Figure 4 | Number of photons with respect to lateral and axial precision.** 3D surface plot of a bead sample shows the dependency of the number of photons with respect to the lateral and axial localization precision.

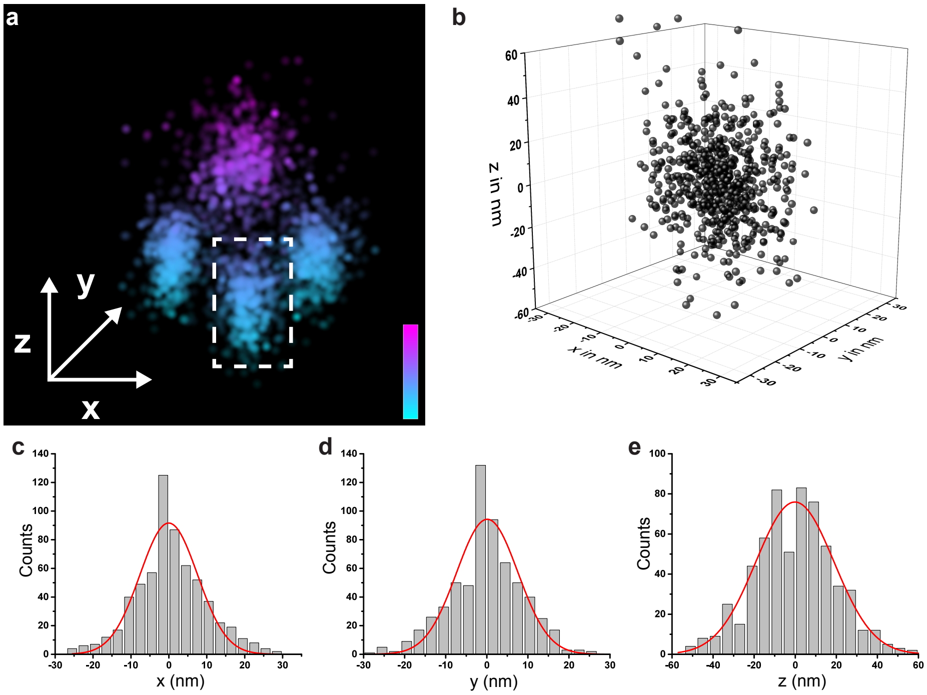


**Supplementary Figure 5 | Assessing x, y, and z localization precision. (a)** Summed tetrahedron image from **Fig. 1f**. The dashed box depicts the corner being analyzed. **(b)** 3D scatter plot of the highlighted corner from (**a**). (**c–e**) Histograms of one-dimensional projections for each dimension separately: $\sigma_x$ = 8 nm, $\sigma_y$ = 8 nm, $\sigma_z$ = 19 nm.

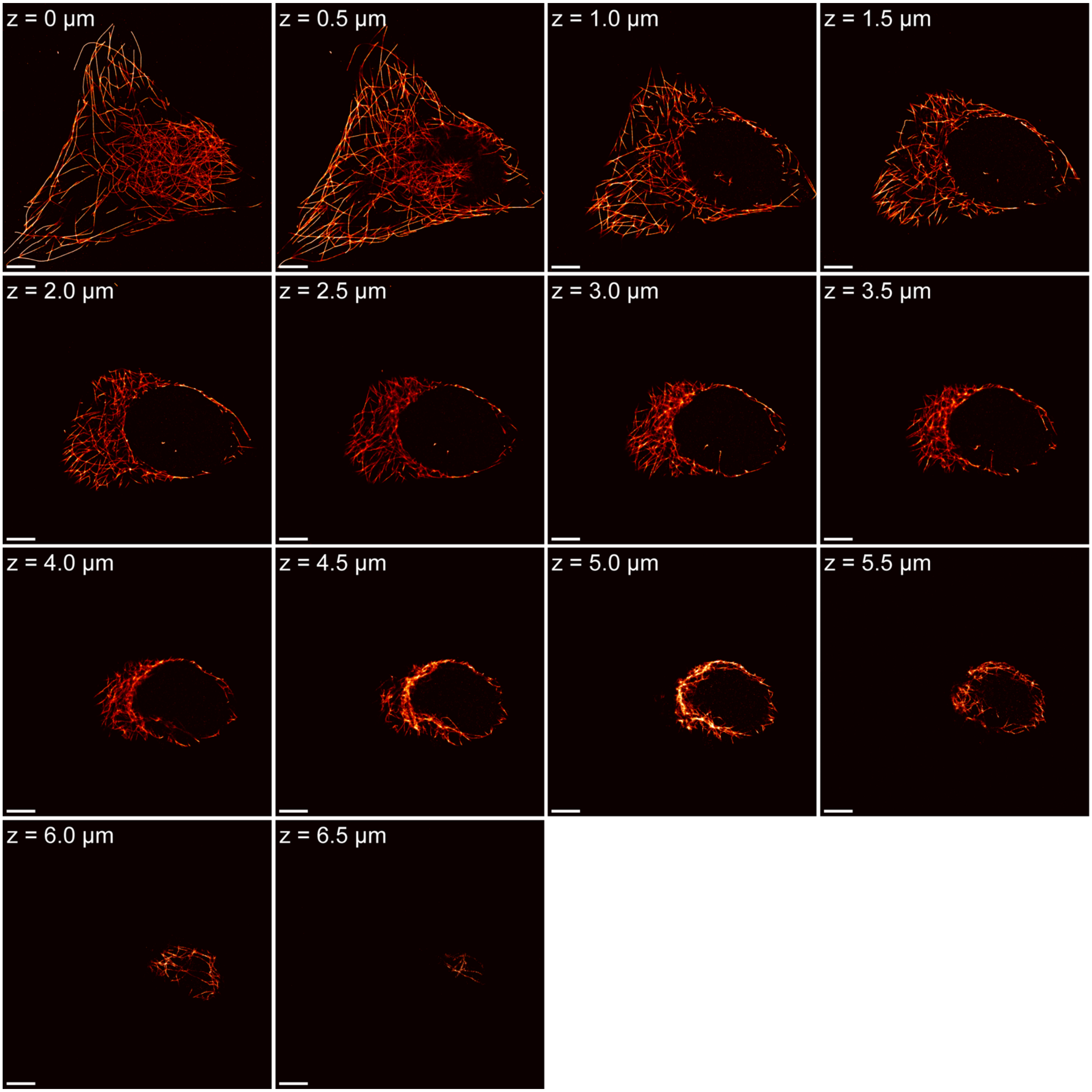


**Supplementary Figure 6 | 500 nm thick slices of the microtubule network through a whole HeLa cell**. The complete stack from **Fig. 2 b–d**. The labeled protein is Alpha-Tubulin. Scale bars, 5 μm.

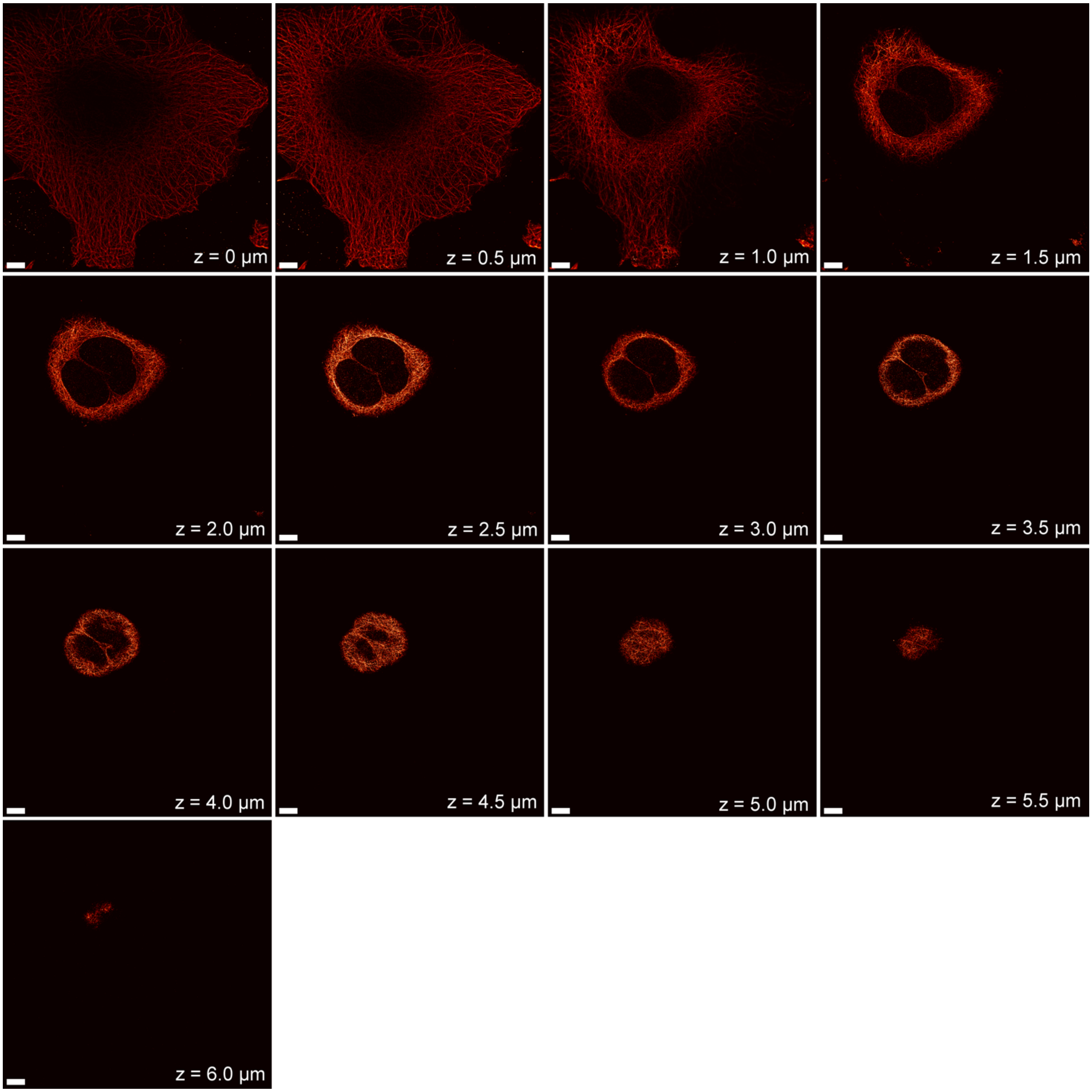


**Supplementary Figure 7 | 500 nm slices of the microtubule network through a whole HeLa cell using a 60x 1.27NA water lens.**
Scale bars, 5 μm. (NeNA values: @ 0 μm: 23.8 nm, @ 2.5 μm: 25.1 nm, @ 5 μm: 23.8 nm)

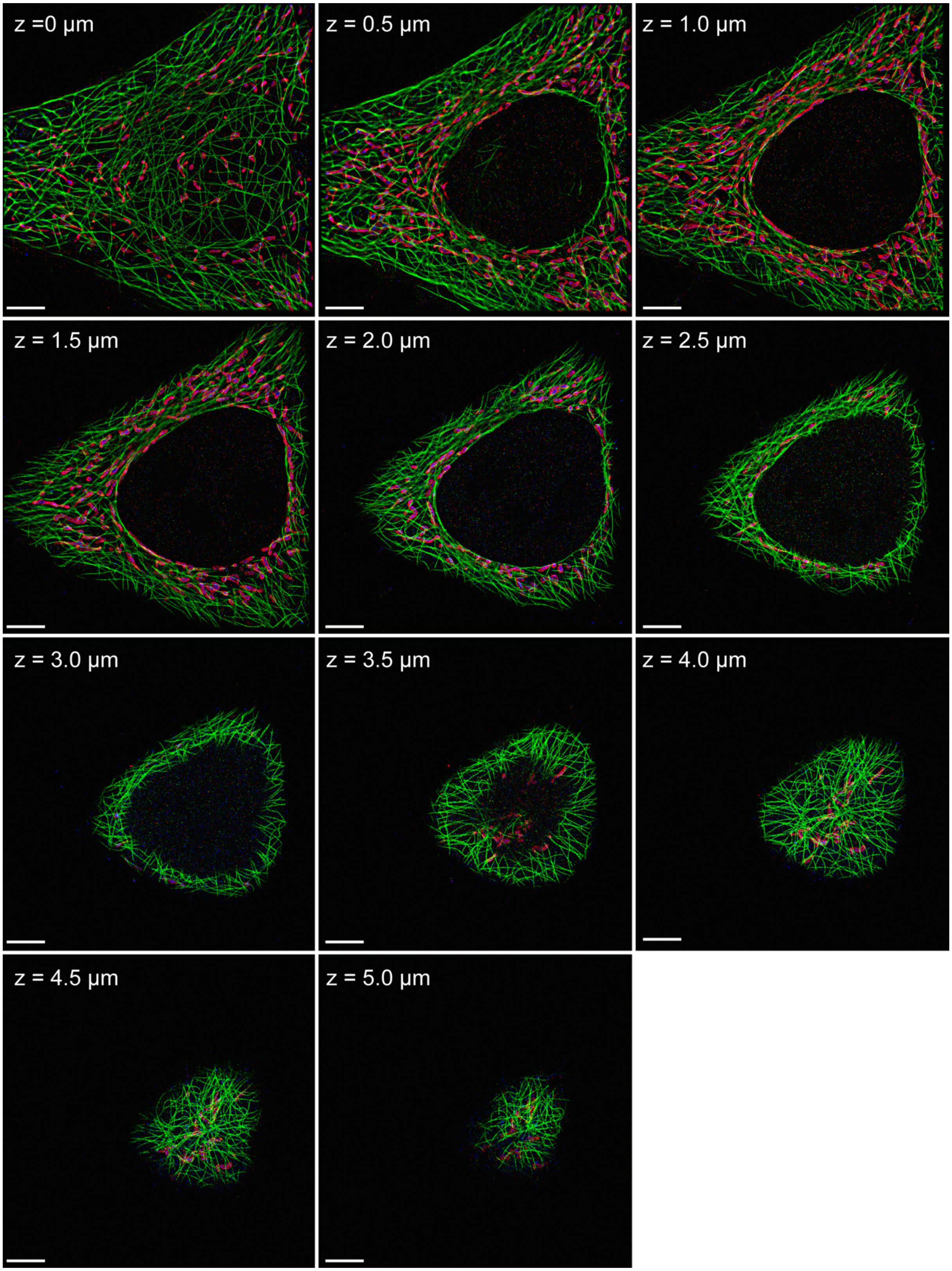


**Supplementary Figure 8 | Three-color stack through a whole HeLa cell.** Complete stack of the three-target (acquired sequentially with Exchange-PAINT) image of **Fig. 2 e–g**. Green: Alpha-Tubulin, Red: TOM20 and Blue: HSP60. Scale bars, 5 μm. (Average 3-target NeNA values: @ 0 μm: 17.2 nm, @ 2.5 μm: 18.3 nm, @ 5 μm: 19 nm)

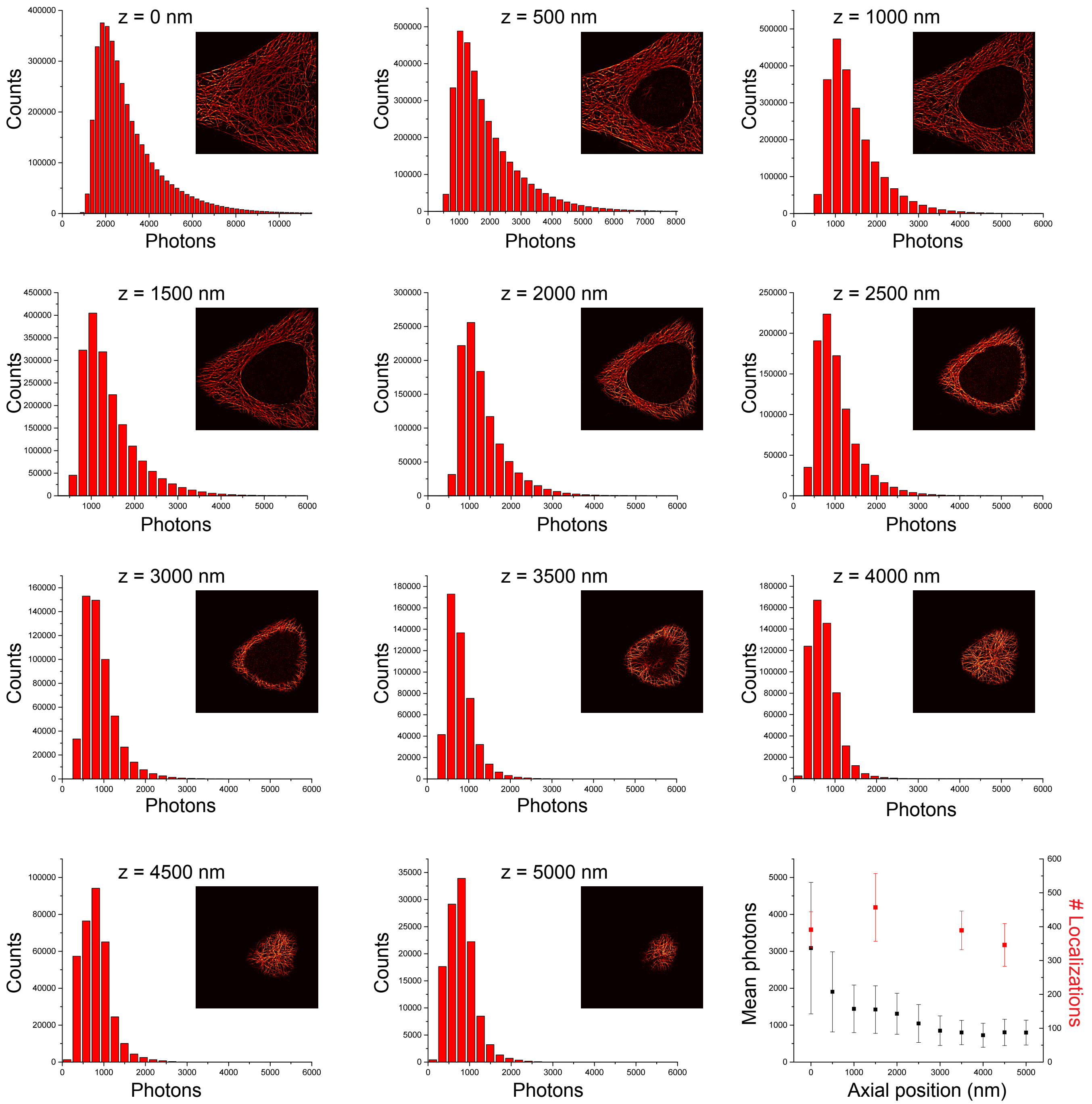


**Supplementary Figure 9 | Distribution of detected photons and localizations at different z-positions.** The number of detected photons decreases with increasing z-position (z = 0 nm = image plane at the coverslip) of the image acquisition plane. Plotted are distributions for z = 0 nm to z = 5000 nm in 500 nm steps along with the mean number of photons (± standard deviation, n>100000) vs. axial position (bottom right, black) and number of detected localizations (± standard deviation, n>1000) vs. axial position (bottom right, red, at z = 0, 1500, 3500, and 4500 nm obtained as average from 10 random pick areas with 320 nm diameter along microtubules per plane).

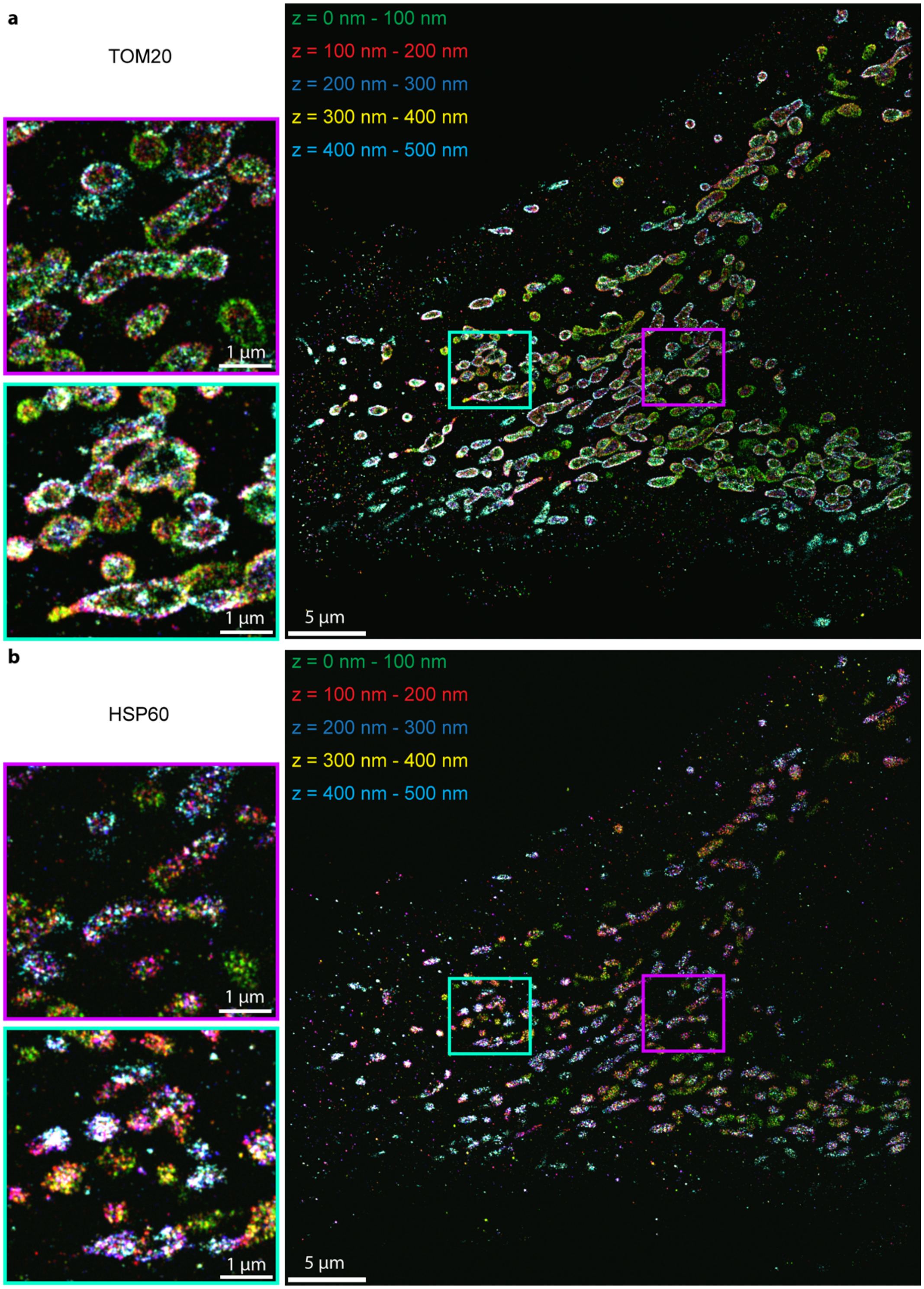


**Supplementary Figure 10 | 3D super-resolved stack of TOM20 and HSP60, 3 µm away from the surface**. **(a)** 500-nm thick super-resolution slice of TOM20 divided into sub-slices of 100 nm labeled by color. The magenta and green framed images are zoom-ins of the highlighted area of the image on the right side. **(b)** Same depiction for HSP60.

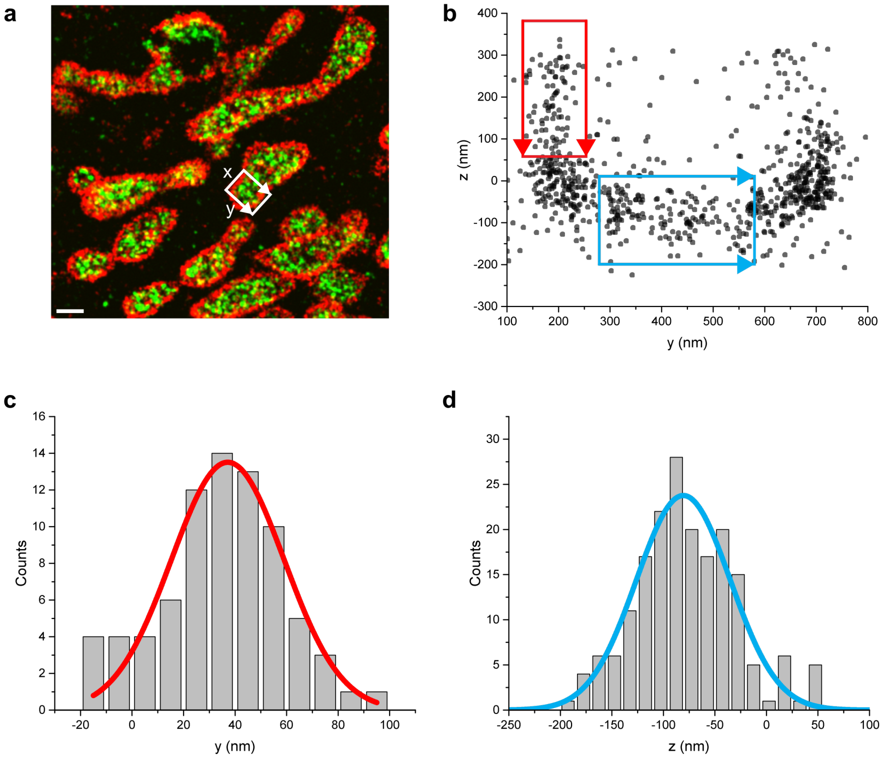


**Supplementary Figure 11 | Assessing 3D resolution at 3 µm inside a cell.** (**a**) 2D projection of three-dimensional data set of the mitochondrial network. TOM20 (red) and HSP60 (green). (**b**) yz-scatterplot of localizations within the highlighted area in (**a**). (**c**) Cross-sectional histogram of the red highlighted area in (**b**)**.** Standard deviation of Gaussian fit (red) is $\sigma_y$ = 20 nm. (**d**) Cross-sectional histogram of the blue highlighted area in (**b**). Standard deviation of Gaussian fit (blue) is $\sigma_z$ = 40 nm. Scale bar, 500 nm.

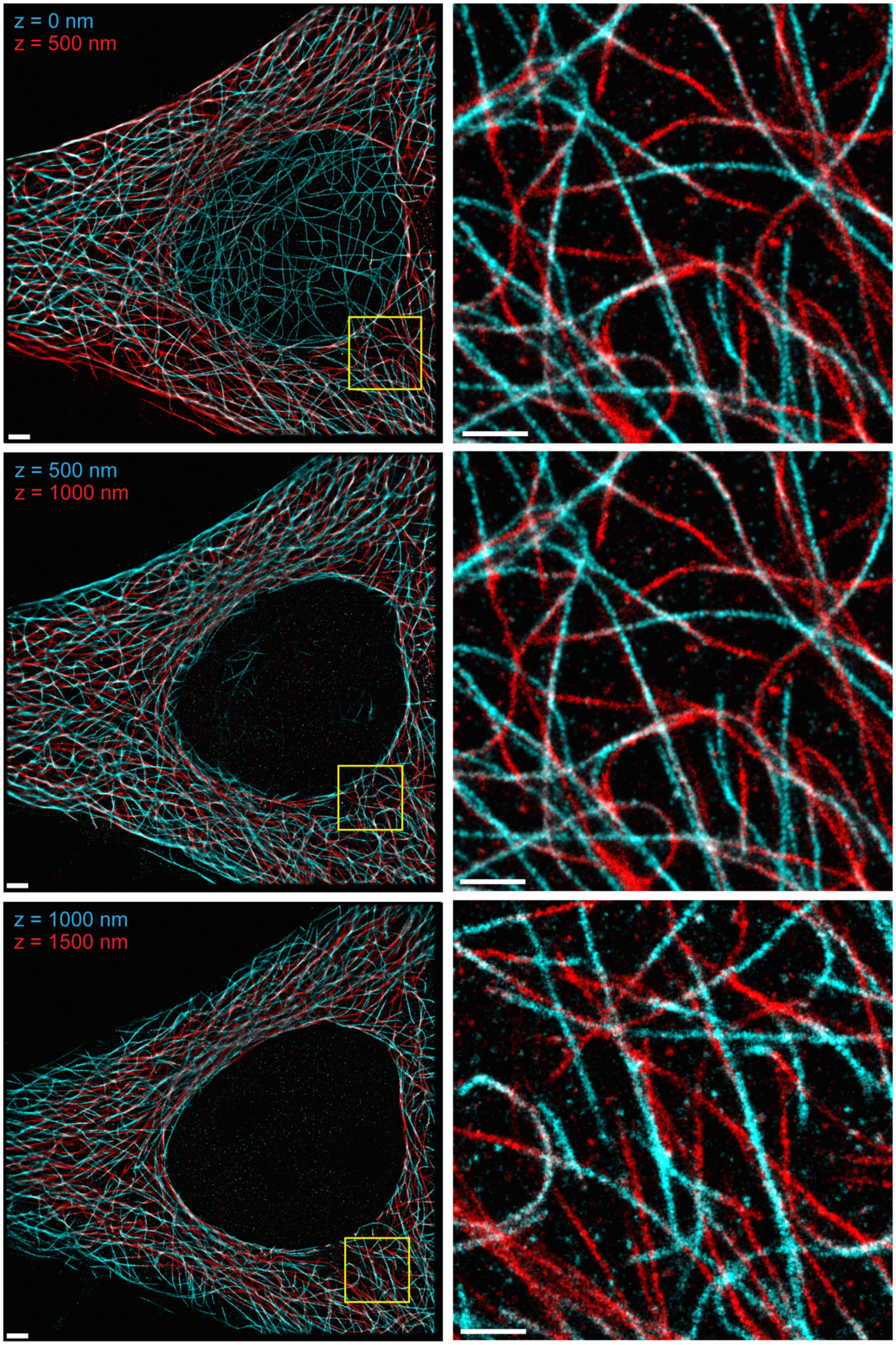


**Supplementary Figure 12 | Overlap of consecutive sections from Fig. 2, z=0–1500 nm.** False-color overlay of consecutive SDC sections from Figure 2. Each pair is acquired 500 nm apart. The lower section is colored cyan and the upper section is colored red. Zoom-ins highlight continuous sampling of microtubules across section borders. Scale bars, 2 μm (left, overviews), 1 μm (right, zoom-ins).

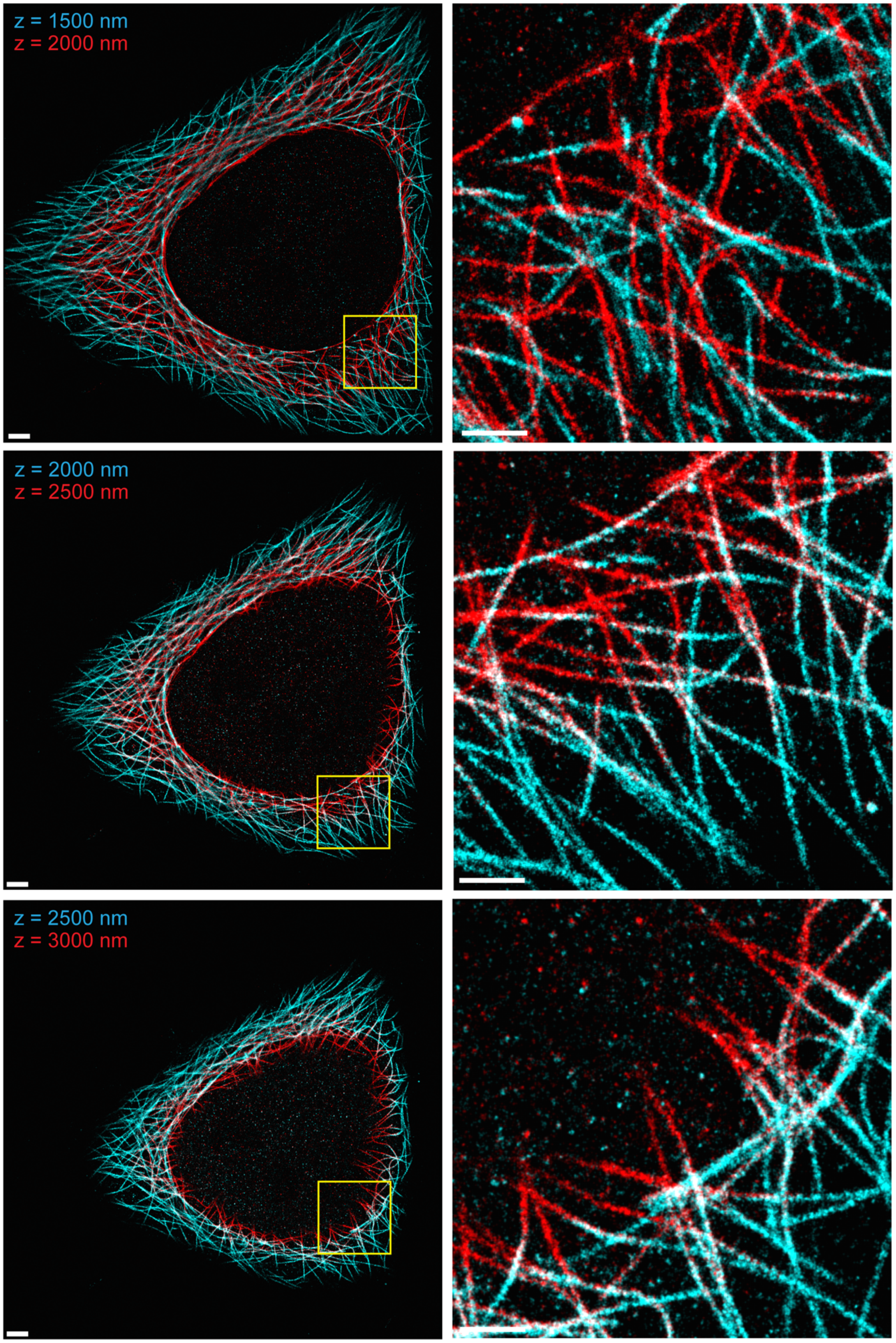


**Supplementary Figure 13 | Overlap of consecutive sections from Fig. 2, z=1500–3000 nm.** False-color overlay of consecutive SDC sections from Figure 2. Each pair is acquired 500 nm apart. The with lower section is colored cyan and the upper section is colored red. Zoom-ins highlight continuous sampling of microtubules across section borders. Scale bars, 2 µm (left, overviews), 1 µm (right, zoom-ins).

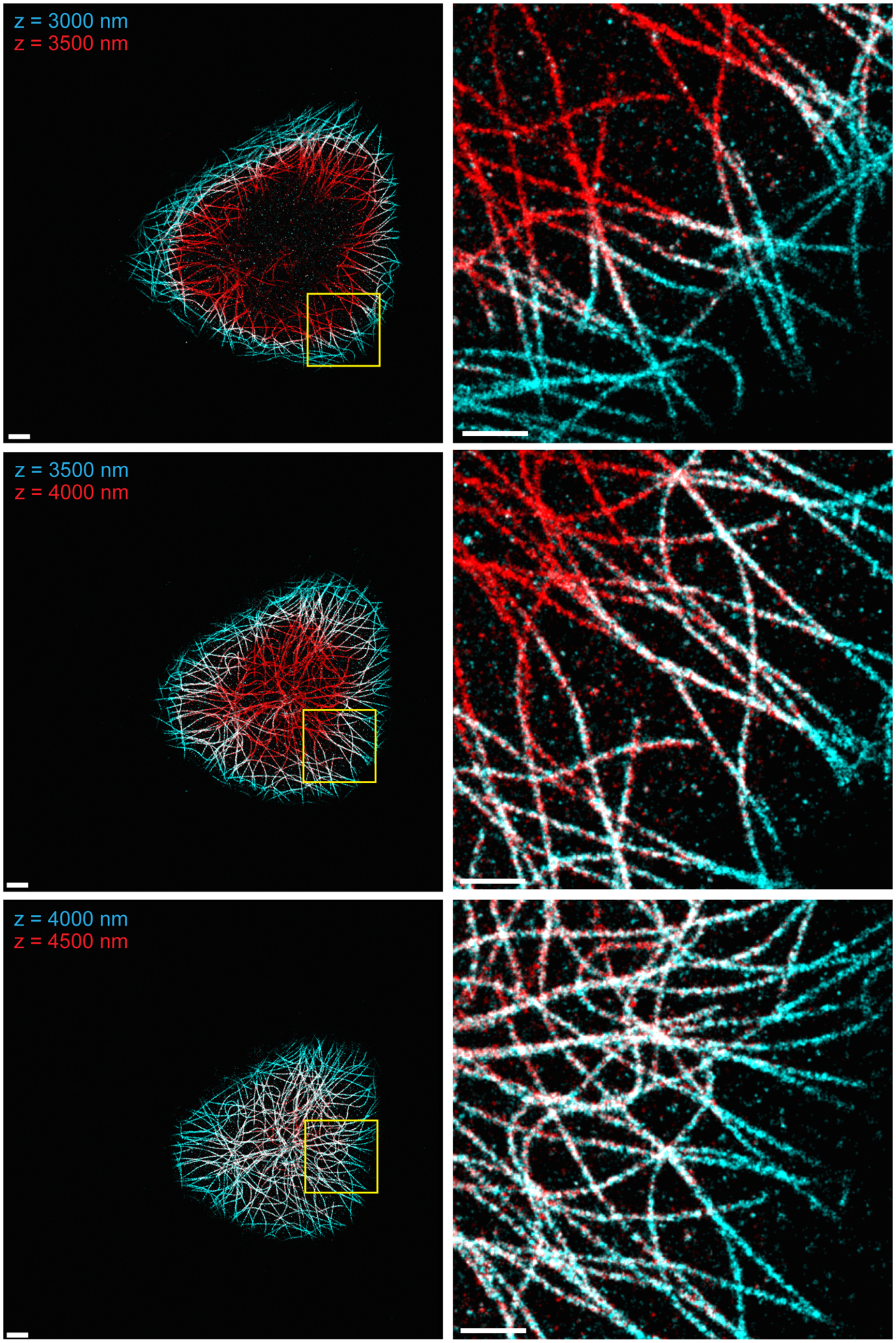


**Supplementary Figure 14 | Overlap of consecutive sections from Fig. 2, z=3000–4500 nm.** False-color overlay of consecutive SDC sections from Figure 2. Each pair is acquired 500 nm apart. The with lower section is colored cyan and the upper section is colored red. Zoom-ins highlight continuous sampling of microtubules across section borders. Scale bars, 2 μm (left, overviews), 1 μm (right, zoom-ins).

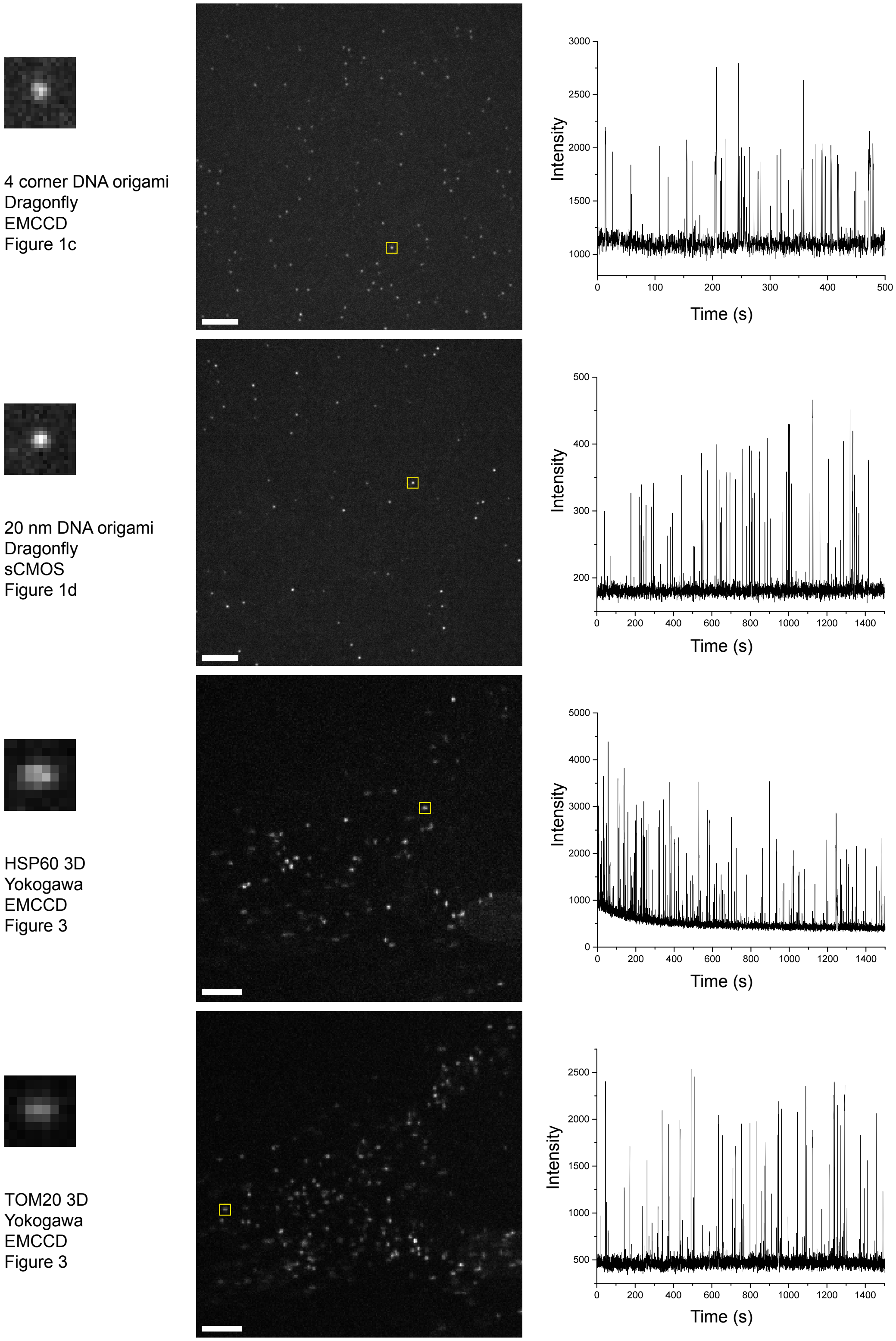


**Supplementary Figure 15 | Raw data of origami and protein target imaging.** Left column presents information about sample, SDC unit, camera and corresponding figure with super-resolution data alongside an exemplary single point-spread-function. Middle column shows a representative raw image frame from the acquisition. Right column shows representative intensity vs. time trace for a diffraction limited area. Scale bars, 5 µm

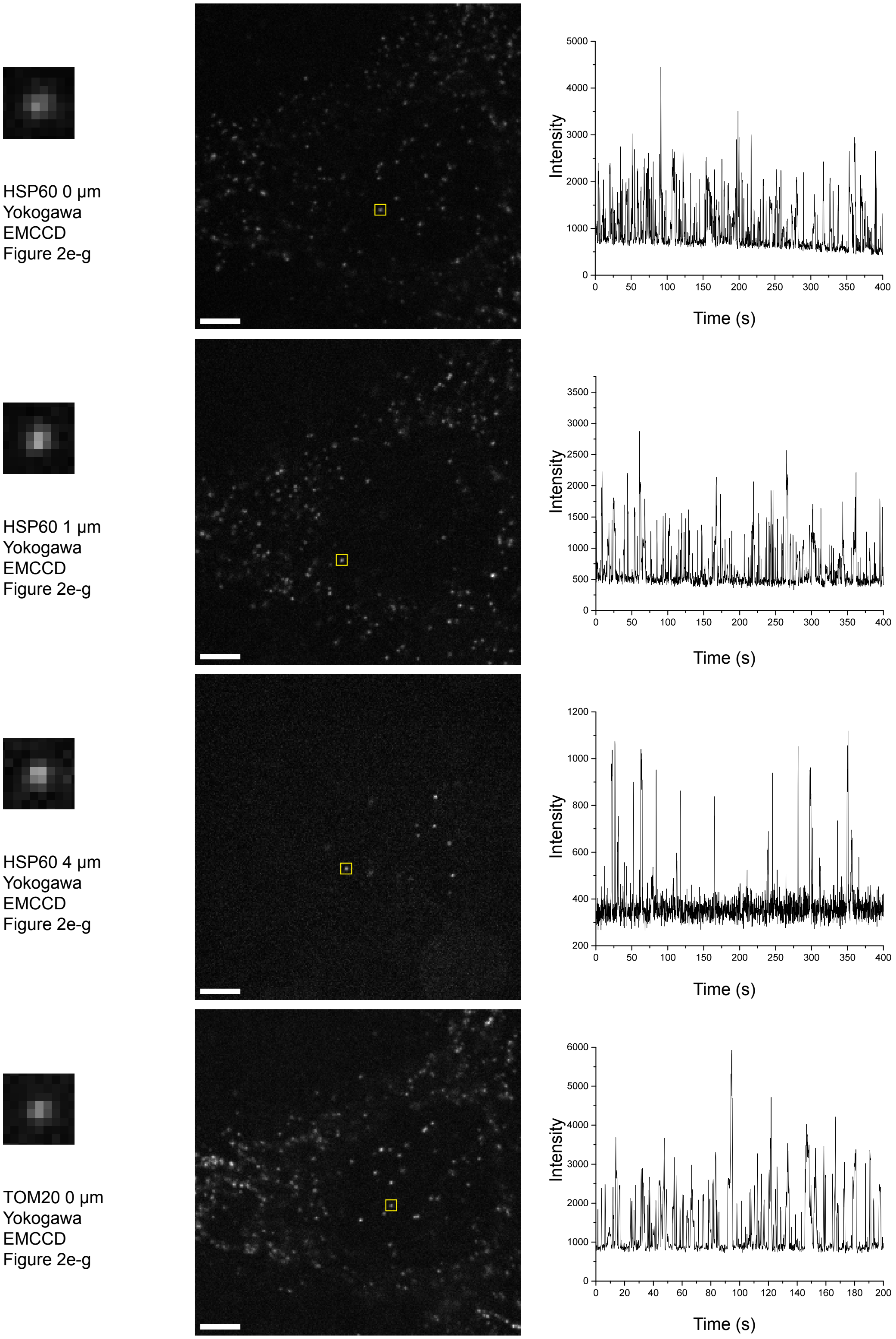


**Supplementary Figure 16 | Raw data of HSP60 and TOM20 imaged at different z positions.** Left column presents information about sample, SDC unit, camera and corresponding figure with super-resolution data alongside an exemplary single point-spread-function. Middle column shows a representative raw image frame from the acquisition. Right column shows representative intensity vs. time trace for a diffraction limited area. Scale bars, 5 µm

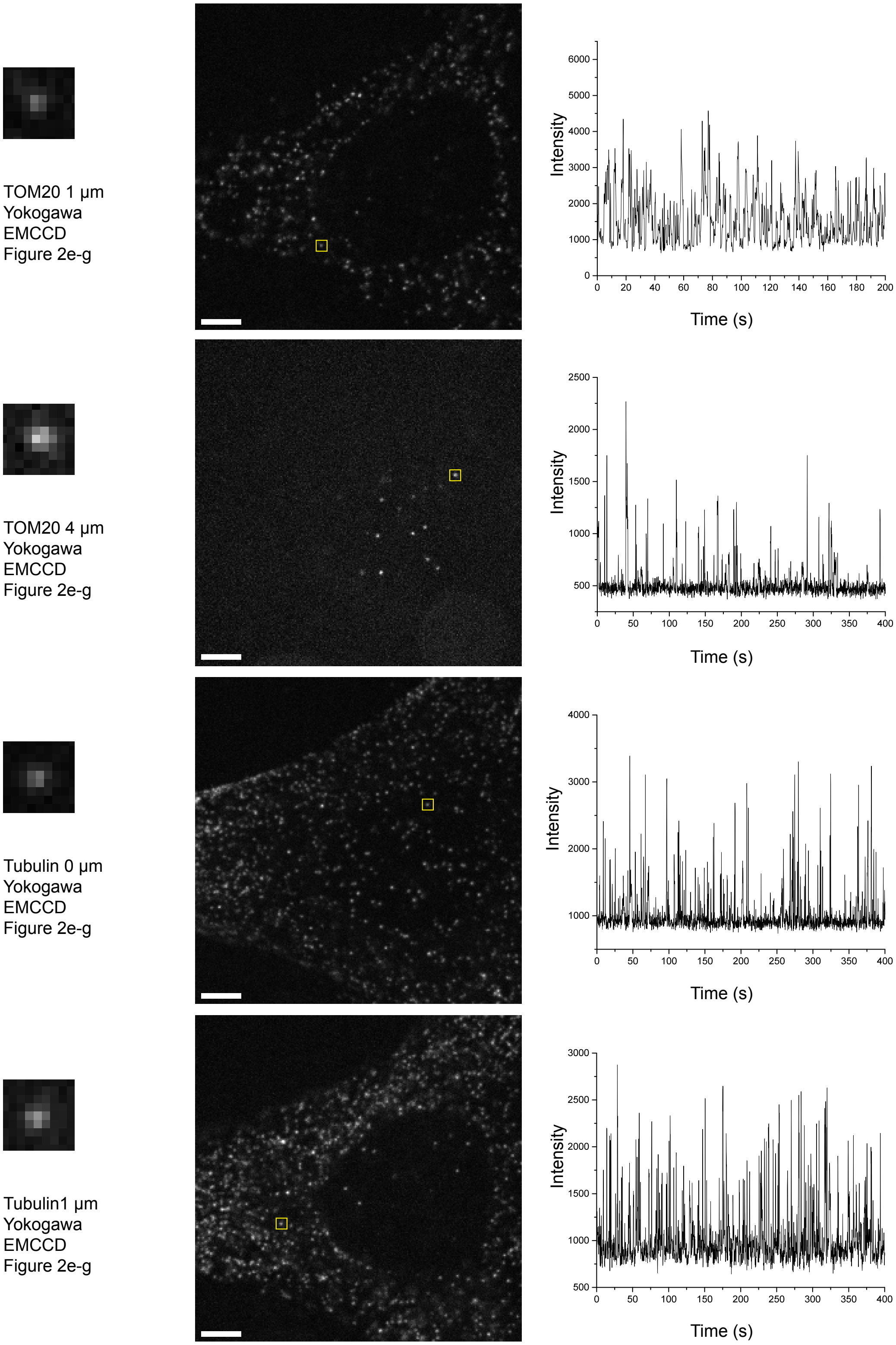


**Supplementary Figure 17 | Raw data of TOM20 and Tubulin imaged at different z positions.** Left column presents information about sample, SDC unit, camera and corresponding figure with super-resolution data alongside an exemplary single point-spread-function. Middle column shows a representative raw image frame from the acquisition. Right column shows representative intensity vs. time trace for a diffraction limited area. Scale bars, 5 µm

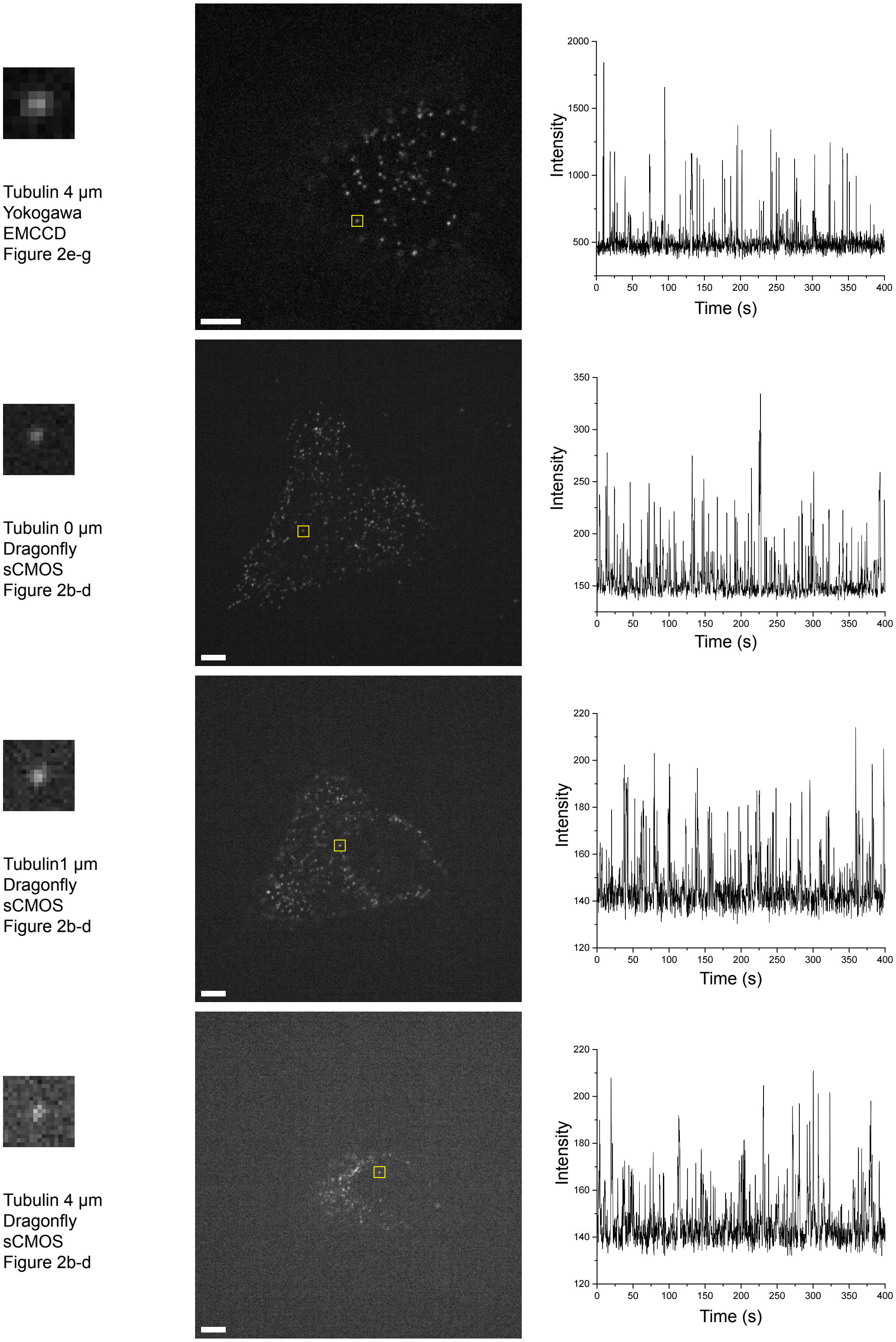


**Supplementary Figure 18 | Raw data of tubulin imaged at different z positions.** Left column presents information about sample, SDC unit, camera and corresponding figure with super-resolution data alongside an exemplary single point-spread-function. Middle column shows a representative raw image frame from the acquisition. Right column shows representative intensity vs. time trace for a diffraction limited area. Scale bars, 5 µm

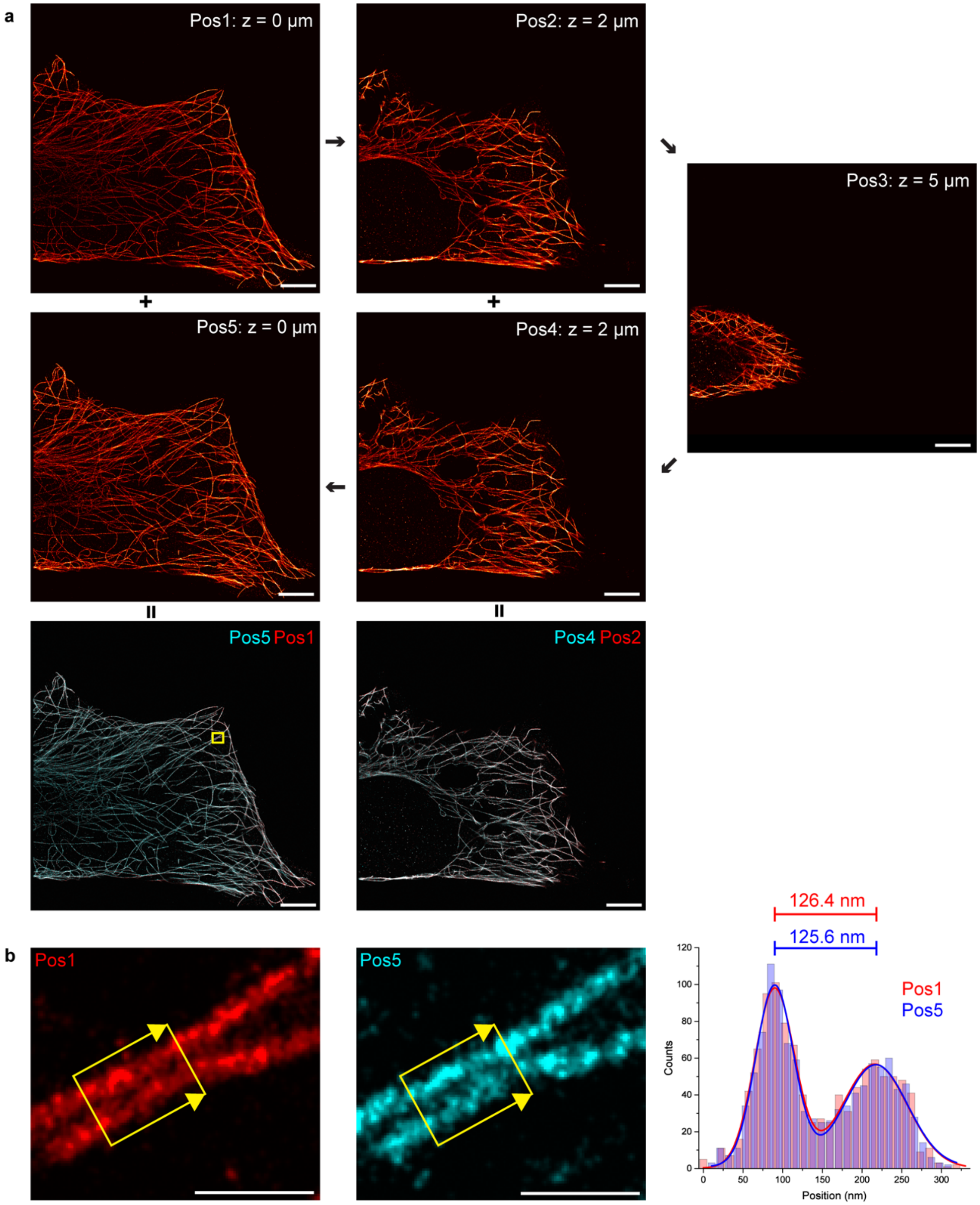


**Supplementary Figure 19 | Position reproducibility experiment**. (**a**) Five z planes were imaged. Starting with the surface (0 μm) going up to 2 μm, next to 5 μm and then back to 2 μm and finally to the surface (0 μm) again (arrows indicate temporal order). The left image in the bottom row shows an overlap of position 1 (red) and position 5 (cyan). The right image in the bottom row depicts an overlap of position 2 (red) and position 4 (cyan). Reproducibility was quantitatively assessed by calculating a normalized cross-correlation coefficient of the image obtained at Pos5 and Pos1 as well as Pos4 and Pos2, respectively. The cross-correlation coefficient was determined to be 0.93 and 0.95 for Pos5-Pos1 and Pos4-Pos2, respectively. (**b**) Zoom in of highlighted area in (**a**) of the overlay image of Pos1 and Pos5. Cross-sectional histogram of two microtubules from the highlighted area (projection along the arrows) for Pos1 (red) and Pos5 (blue/cyan). The distance deviation of Pos1 to Pos5 is ~ 1nm. Scale bars, 5 μm (**a**), 500 nm (**b**).

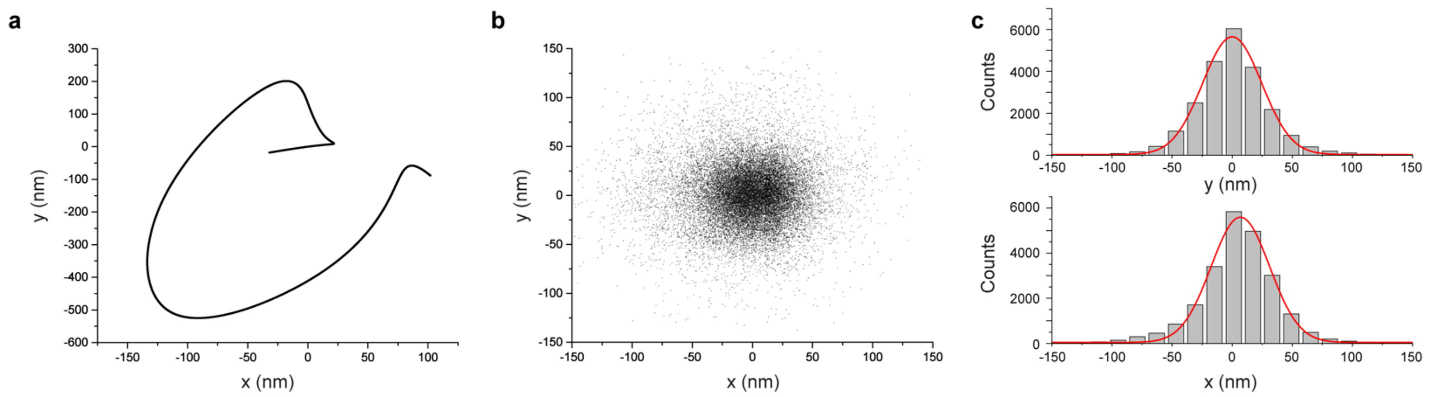


**Supplementary Figure 20 | Performance of Redundant Cross Correlation (RCC) drift correction.** (**a**) Calculated x-y drift correction using RCC for data from **Fig. 4a** (Lamin B). (**b**) X- and y-positions of a 40-nm bead post-drift-correction from the same sample. Resulting localization distributions show no detectable 'skewing' due to residual drift. (**c**) Calculated standard deviations from localizations in (**b**): x = 24.33 nm, y = 24.55 nm.

**Supplementary Table 1 |** Localization precision of each plane of **Fig. 2 b–d** and **Supplementary Figure 6** as determined by the Cramér-Rao lower bound (CRLB) of the single-molecule fit.

| Z position | CRLB |
|---|---|
| 0.0 µm | 9.8 nm |
| 0.5 µm | 11.7 nm |
| 1.0 µm | 12.6nm |
| 1.5 µm | 13.3 nm |
| 2.0 µm | 13.7 nm |
| 2.5 µm | 15.1 nm |
| 3.0 µm | 15.6 nm |
| 3.5 µm | 15.9 nm |
| 4.0 µm | 16.1 nm |
| 4.5 µm | 16.3 nm |
| 5.0 µm | 16.8 nm |
| 5.5 µm | 18.3 nm |
| 6.0 µm | 17.8 nm |
| 6.5 µm | 21.1 nm |

**Supplementary Table 2 |** Average localization precision of each plane and target of **Fig. 2 e–g** and **Supplementary Figure 8** as determined by CRLB.

| | Alpha-Tubulin | TOM20 | HSP60 |
|---|---|---|---|
| Z position | CRLB | CRLB | CRLB |
| 0.0 µm | 7.5 nm | 11.1 nm | 11.7 nm |
| 0.5 µm | 10.4 nm | 10.1 nm | 10.4 nm |
| 1.0 µm | 10.2 nm | 10.3 nm | 11.9 nm |
| 1.5 µm | 10.5 nm | 10.9 nm | 12.1 nm |
| 2.0 µm | 10.7 nm | 11.2 nm | 12.2 nm |
| 2.5 µm | 12.3 nm | 12.4 nm | 12.7 nm |
| 3.0 µm | 12.4 nm | 12.8 nm | 14.5 nm |
| 3.5 µm | 12.7 nm | 15.8 nm | 15.0 nm |
| 4.0 µm | 12.9 nm | 13.7 nm | 17.4 nm |
| 4.5 µm | 12.6 nm | 12.6 nm | 16.8 nm |
| 5.0 µm | 15.4 nm | 15.0 nm | 19.4 nm |

**Supplementary Table 3 |** DNA handle extensions and imager strand sequences.

| Name (length) | Sequence | Corresponding Antibody/Structure |
|---|---|---|
| P1 anti-handle | 5′ - CTAGATGTAT - dye - 3′ | |
| P2 anti-handle | 5′ - TATGTAGATC - dye - 3′ | |
| P4 anti-handle | 5′ - GTAGATTCAT - dye - 3′ | |
| P5 anti-handle | 5′ - CATACATTGA - dye - 3′ | |
| P15 anti-handle | 5′ - GAATCACTAT - dye - 3′ | |
| Biotin anti-handle (21 nt) | 5′ - biotin - GAATCGGTCACAGTACAACCG - 3' | (Only for Tetrahedron) |
| P1 handle (9 nt) | 5′ - staple - TTATACATCTA - 3′ | Anti-Rat antibody |
| P1 handle (10 nt) | 5′ - staple - TTATACATCTAG - 3′ | |
| P2 handle (10 nt) | 5′ - staple - TTGATCTACATA - 3′ | |
| P4 handle (9 nt) | 5′ - staple - TTATGAATCTA - 3′ | Anti-Rabbit antibody |
| P5 handle (9 nt) | 5′ - staple - TTTCAATGTAT - 3′ | Anti-Mouse antibody |
| P15 handle (10 nt) | 5′ - staple - TTATAGTGATTC - 3′ | |
| Biotin handle (21 nt) | 5′ - staple - TTCGGTTGTACTGTGACCGATTC - 3′ | (Only for Tetrahedron) |

**Supplementary Table 4 |** List of biotinylated staple strands of the RRO structure

| No | Position | Name | Sequence | Mod |
|---|---|---|---|---|
| 1 | C02 | 18[63]20[56]BIOTIN | ATTAAGTTTACCGAGCTCGAATTCGGGAAACCTGTCGTGC | 5'-BT |
| 2 | C09 | 4[63]6[56]BIOTIN | ATAAGGGAACCGGATATTCATTACGTCAGGACGTTGGGAA | 5'-BT |
| 3 | G02 | 18[127]20[120]BIOTIN | GCGATCGGCAATTCCACACAACAGGTGCCTAATGAGTG | 5'-BT |
| 4 | G09 | 4[127]6[120]BIOTIN | TTGTGTCGTGACGAGAAACACCAAATTTCAACTTTAAT | 5'-BT |
| 5 | K02 | 18[191]20[184]BIOTIN | ATTCATTTTTGTTTGGATTATACTAAGAAACCACCAGAAG | 5'-BT |
| 6 | K09 | 4[191]6[184]BIOTIN | CACCCTCAGAAACCATCGATAGCATTGAGCCATTTGGGAA | 5'-BT |
| 7 | O02 | 18[255]20[248]BIOTIN | AACAATAACGTAAAACAGAAATAAAAATCCTTTGCCCGAA | 5'-BT |
| 8 | O09 | 4[255]6[248]BIOTIN | AGCCACCACTGTAGCGCGTTTTCAAGGGAGGGAAGGTAAA | 5'-BT |

**Supplementary Table 5 |** Oligo FISH probes targeting mouse repeats

| Target | Sequence |
|---|---|
| Telomere | GGGTTAGGGTTAGGGTTAGGGTTAGGGTTAGGGTTAGGGTTAGGGTTAttATACATCTAG |
| Minor Satellite | AGATGAGTGAGTTACACTGAAAAACACATTCGTTGGAAACGGttGATCTACATA |
| Major Satellite (Fig. 4b) | GCGAGGAAAACTGAAAAAGGtttATACATCTA |
| Major Satellite (Fig. 4c) | CCACTGTAGGACGTGGAATATGGCAAGAAAACTGAAAATCATGGttATAGTGATTC |

**Supplementary Table 6 |** Oligopaint probe sets used

| Target | Homology Length | Assembly | Chr. | Span (kb) | Start | Stop | Complexity | Probes /kb |
|---|---|---|---|---|---|---|---|---|
| Xq28 Probe 1 | 40-45 | hg38 | X | 817 | 153051165 | 153868151 | 4,776 | 5.9 |
| Xq28 Probe 2 | 40-45 | hg38 | X | 1831 | 153868207 | 155699694 | 5,509 | 3.0 |
| Xist lncRNA | 36-41 | hg19 | X | 11.4 | 73841382 | 73852735 | 167 | 14.7 |
| CBX5 mRNA | 28 | hg19 | 12 | 49.1 | 54624816 | 54673870 | 162 | 3.3 |
