# Supplementary material for: Multiplexed 3D super-resolution imaging of whole cells using spinning disk confocal microscopy and DNA-PAINT
Source: Nat Commun. 2017 Dec 12;8:2090. doi: 10.1038/s41467-017-02028-8 (PMC5727263; doi:10.1038/s41467-017-02028-8)
Supplement: Supplementary file 3 — Description of Additional Supplementary Files [file 41467_2017_2028_MOESM3_ESM.pdf]

Descriptions of Additional Supplementary Files

File Name: Supplementary Data 1
Descriptions: List of staple strands of the DNA origami tetrahedron structure

File Name: Supplementary Data 2
Descriptions: List of staple strands for the RRO structure. Yellow marked staples were extended with a P1, 9 nt sequence (Supplementary Table 3) handle in the “4 corner” structure. Green marked staples were extended with a P1, 9 nt sequence (Supplementary Table 3) in the 3 x 4 ‘20 nm grid’ structure.

File Name: Supplementary Data 3
Descriptions: Sequence of the M13mp18 scaffold

File Name: Supplementary Data 4
Descriptions: Sequence of the p8064 scaffold

File Name: Supplementary Movie 1
Descriptions: TOM20 + HSP60, 3D DNA-PAINT, 3 µm inside a HeLa cell

File Name: Supplementary Movie 2
Descriptions: TOM20, 3D DNA-PAINT, 3 µm inside a HeLa cell. XZ-view of mitochondria network (slice 3 µm away from the coverslip). Slicing along the y axis with 100 nm thickness in y. The movie clearly shows (depending on the position and thickness of the mitochondria relative to the slice) mitochondria open at top, at the bottom and also closed ring-like mitochondria.

File Name: Supplementary Movie 3
Descriptions: Raw Data of 3D imaging. Movie with 1000 raw images each recorded with 200 ms exposure time of the 3D TOM20 sample 3 µm away from the coverslip.
